# Construction of a police physical evaluation model and standards based on law enforcement ability

**DOI:** 10.3389/fphys.2023.1330371

**Published:** 2024-01-08

**Authors:** Yuliang Feng, Yang Liu, Dexin Wang

**Affiliations:** ^1^ School of Athletic Performance, Shanghai University of Sport, Shanghai, China; ^2^ Police Training Department, Shanghai Police College, Shanghai, China; ^3^ School of Physical Education, Nanchang Normal University, Nanchang, Jiangxi, China

**Keywords:** physical training, police law enforcement, evaluation index, criteria, tactical

## Abstract

**Purpose:** In the context of police practical combat with the essence of coercion and confrontation, police physical fitness training guided by practical combat is increasingly valued. The objective of this study was to establish a police physical evaluation model and standards based on law enforcement ability.

**Methods:** Using literature analysis, expert interviews, Delphi method, field testing, and mathematical statistics, the test results of 301 frontline law enforcement police officers from the Shanghai Public Security Bureau were used as sample data. Factor analysis was conducted on the selected indicators to determine the final indicator system for police physical fitness. The percentile method was used to develop evaluation standards, and frequency statistics and chi-squared tests were used to calculate the effectiveness and accuracy of the evaluation standards.

**Results:** The research results revealed that the police physical fitness evaluation model based on law enforcement ability is Y = 0.115 × 30 s of trigger pulling according to the gun + 0.105 × 30 s of straight punching sandbags + 0.095 × 30 s of wrestling the dummy + 0.062 × push-ups + 0.115 × 50-m run + 0.095 × 10 m × 4 round trips + 0.116 × standing long jump + 0.090 × 2,000-m run + 0.089 × 30 s of hitting sandbags using a short baton + 0.118 × 30 s of poking sandbags using the long baton. The evaluation criteria were divided into single-item and comprehensive evaluation criteria.

**Conclusion:** A practical police physical fitness evaluation model can effectively evaluate the level of police physical fitness development, and the evaluation standards can provide an effective basis for a practical police physical fitness evaluation.

## Introduction

Police officers must be physically fit to perform all the requirements of their profession and to maximize the safety of the community and the people involved; consequently, physical fitness is a non-negligible part of a police officer’s ability to accomplish the tasks of law enforcement work (Bonneau and Brown, 1995). Studies have shown that the physical fitness level of police officers has declined in recent years (Orr et al., 2018), while the current changes in law enforcement confrontation situations have presented new challenges to police physical fitness, and carrying and using relevant police-specialized equipment to accomplish unpredictable law enforcement tasks, like controlling, lifting, dragging, and pursuing a suspect, require a higher physical output (Ehnes et al., 2020). Consequently, police training based on law enforcement ability has become increasingly important. Law enforcement ability is where police officers use coercion as the main form of clearing confrontation (Zhang Bing, 2006), including escape and pursuit, control and counter-control, and even combat and sparring; thus, coercion and confrontation are the essential characteristics of police combat (Liu Kaiji, 2014). The evaluation model is a quantitative evaluation template and a part of the evaluation system (Zhang et al., 2021), while an evaluation system plays a guiding role in training. The current police physical quality evaluation indicators, including the content of the indicators, are no different to those of ordinary people, focusing mostly on general quality and non-specific confrontation quality indicators (Dong Rujun, 1999; Tian wen, 2008; Chen, 2009). However, the tasks that police officers need to complete—that is, police combat in a police active attack, passive counterattack, or return attack—usually do not change with the increasing age of the officers: the suspects are undifferentiated and the level of aggressiveness and the number of assaults are not reduced as the officers age. Police physical training under the law enforcement confrontation situation requires a focus on actual combat and should organically combine police physical fitness with police technical and tactical training to show its role and practical significance (Liu and Shi, 2014; Luo Weidong, 2014). Therefore, there is an urgent need to build a unified, objective, and practical police physical fitness evaluation system in the police sector based on law enforcement ability. The purpose of this study was to determine the police physical fitness evaluation index system, calculate and construct the physical fitness evaluation model, establish the corresponding evaluation criteria based on the police law enforcement ability, combine physical fitness and equipment skills, and provide new ideas for police physical training.

## Materials and methods

### Expert opinions

***Literature analysis:*** Information about police physical fitness training and evaluation at home and abroad was collected from the Web of Science, PubMed, EBSCO, China Knowledge Network, and other databases as theoretical support. The search terms used were as follows: “police” OR “law enforcement” AND “physical fitness” AND “training” OR “strength and conditioning” AND “evaluation.” There were no limitations to the start date in this study; until 2022, a total of 106 relevant literature works were obtained. ***Expert interview:*** The selected experts were mainly engaged in academic research on physical fitness training, practical research on police physical training in police colleges, and police frontline unit leaders or backbones. The contents of the interview included the following questions: what kind of physical fitness and equipment-use skills are needed in police law enforcement tasks? What are the shortcomings of the existing evaluation indicators? How to combine physical fitness with skill to construct an evaluation index system based on police law enforcement ability? In-depth interviews and exchanges were conducted to obtain expert suggestions on the trade-offs between the existing evaluation indicators of police physical fitness and the combat-oriented evaluation indicators of police physical fitness. ***Delphi method:*** The initial indicators were distributed to 13 experts in the above-mentioned fields, and two rounds of consultation were conducted to achieve a convergence of expert opinions. The questionnaire was designed according to the degree of importance (given as 5, 4, 3, 2, and 1, respectively) to screen and determine the quality evaluation indexes of police upper body confrontation, lower body confrontation, and equipment confrontation under the law enforcement orientation. After two rounds of concentrated opinions and screening, a police physical quality evaluation index system was constructed.

Opinion concentration (*M*_*j*_) is the arithmetic mean of the scores obtained for each indicator; opinion coordination (*V*_*j*_) is the coefficient of the variation of the scores obtained for each indicator. The higher the concentration of opinions, the smaller the coefficient of variation and the higher the recognition of the indicators by experts.

$$\text{Mj}=\frac{1}{n}\sum_{i=1}^{n}X\text{ij}.$$ (1)

Suppose *X*_*ij*_ denotes the score of the *i*th expert for the *j*th indicator with *n* experts and *m* indicators, then

$$\text{Sj}=\sqrt{\frac{1}{n-1}\sum_{i=1}^{n}\left(Xij-Mj\right)²}.$$ (2)

The formula for calculating the coefficient of variation is *V*_*j*_ *= S*_*j*_/*M*_j_, where *V*_*j*_ is the coefficient of the variation of indicator *j*, *S*_*j*_ is the standard deviation of indicator *j*, and *M*_*j*_ is the arithmetic mean of indicator *j.* A smaller *V*_*j*_ value indicates a higher degree of expert opinion coordination for indicator *j* (Tian Jun et al., 2004).

By reviewing and analyzing the literature related to police physical training and physical quality evaluation (Dong Rujun, 1999; Tian Wen, 2008; Chen, 2009; Marins et al., 2019), collecting the commonly used police physical quality evaluation indexes, and combining the results of expert interviews, we initially screened the physical quality evaluation indexes that are closely related to and representatives of police law enforcement combat. These indexes comprised three primary indexes, namely, upper limb confrontation, lower limb confrontation, and police equipment confrontation quality, and 25 secondary indexes.

In this study, the concentration of expert opinions is greater than 4.0, and the coordination degree is less than 0.2. According to the Eqs 1, 2, three primary indicators, namely, upper body confrontation, lower body confrontation, and police equipment confrontation quality, and 13 secondary indicators, namely, push-ups (4.40), standing long jump (4.21), 50-m run (4.16), 2,000-m run (4.02), 10 m × 4 round-trip run (4.02), sitting forward bending (4.00), 30 s of straight punching sandbags (4.20), 30 s of kicking sandbags (4.16), 30 s of wrestling the dummy (4.40), 30 s of hitting sandbags using the short baton (4.08), 30 s of poking sandbags using the long baton(4.16), 30 s of pushing sandbags using the shield (4.20), and 30 s of trigger pulling according to the gun linkage (4.05), were used.

### Participants

All participants provided their written informed consent. The survey subjects were assured confidentiality and anonymity of the collected data. From September 2022 to December 2022, using a whole-group sampling method, a team of uniformly trained Shanghai police physical fitness instructors conducted physical fitness tests for the training rotation of public security police officers in 16 district public security bureaus in Shanghai. The inclusion criteria for this analysis were as follows: 1) voluntary participation and good health; 2) male civilian police officers engaged in frontline law enforcement work (except SWAT police officers); and 3) not being deployed for prolonged security or major riot control tasks 3 days before the test. The exclusion criteria were as follows: 1) recently injured or suffering from diseases that may affect the physical fitness test; 2) SWAT police and female civilian police officers; and 3) having participated in a long period of security or riot control work that may cause fatigue before the test. Among the 320 police officers in the training rotation groups, 19 were excluded and 301 test subjects were included in the final analysis. The basic characteristics of the test subjects are shown in Table 1.

**TABLE 1:** Basic characteristics of test subjects.

Characteristic	Participating in the civilian police competition (“excellent” group)	General civilian police (“general” group)
Sample size (*n*)	28	273
Age (years)	33.11 ± 5.86	33.57 ± 7.97
Height (m)	1.79 ± 4.73	1.78 ± 3.91
Body weight (kg)	78.55 ± 6.76	77.28 ± 5.83
Length of police service (years)	8.33 ± 0.89	8.81 ± 0.92

*n*, the number of people; m, meter.

The test subjects were divided into “excellent” and “general” groups. The “excellent” group was identified by experts and the police industry and comprised police officers who participated in the police comprehensive physical fitness competition of “all-police combat training” in each public security bureau. The “general” group was the general public security police officers who participated in the rotation training and rotation-duty centralized training. There was no significant difference between the two groups in terms of age, height, weight, and years of police service (*p* >0.05).

### Police physical fitness evaluations

#### Testing instruments

Stopwatch (brand: Casio), standing long jump test mat (brand: Hongkangda), wooden blocks (specifications: 10 cm × 5 cm × 5 cm), dummies (specifications: height: 170 cm; weight: 25 kg), short batons (i.e., telescopic baton; retracted length: 192 ± 1.5 mm; extended length: 412 ± 2 mm; grip outside diameter: 26.5 ± 0.15 mm; mass: ≤290 g), long batons (i.e., riot baton; length: 1,600 mm; stick body outer diameter: 30 mm; weight: 1.24 kg), shield (i.e., riot shield; size: 900 mm × 500 mm × 3.5 mm; weight: 2.5 kg), electronic sandbags (brand: WESING; model: WJXL-QDKP), and nine-two police pistol (full gun length: 190 mm; full gun mass: 0.76 kg; trigger weight: 5 kg) were used in this study.

#### Threshold test

The electronic sandbag hitting thresholds were determined using a random sample of 30 frontline law enforcement police officers of different ages, heights, and weights in a pre-test that involved hitting the electronic sandbag in different ways for 30 s. The average hitting force, in kg and not counting decimal places, was calculated as the threshold value. The measured threshold of straight punching was 40 kg, the threshold of positive stomp was 119 kg, the threshold of short-baton hitting was 69 kg, the threshold of long-baton poking was 66 kg, and the threshold of shield pushing was 56 kg.

#### Testing process

Before the test, a standardized 15-min warm-up was organized for the subjects, which included one set of running, jumping, and dynamic stretching exercises. The test was then conducted in the order of upper limb confrontation, lower limb confrontation, and police equipment confrontation quality, leaving a sufficient interval between each element of the test. In addition, the 2,000-m run was conducted at the end.

#### Test monitoring

1) Action quality monitoring: In each test, action specifications were emphasized to ensure action quality and avoid acute injuries and accidental injuries.
2) Test process monitoring: A standardized site layout plan was developed, and the content and sequence of each test were completed in strict accordance with the principle of maximum space utilization.
3) Test personnel monitoring: Test personnel composed of experienced instructors engaged in police teaching and training, and all instructors had a professional background in sports. Furthermore, the test personnel received unified training in commands, rules, and methods before the test to ensure the smooth conduct of the test.

#### Evaluation protocol

***Push-ups:*** The maximum number of push-ups completed in 1 minute was recorded. ***Standing long jump*:** The maximum jumping distance with both feet forward (from the jump line to the back heel) was recorded. ***50-m run*:** When the test participants heard the start command, they ran 50 m as fast as they could. ***2*,*000-m run*:** The test participants completed five laps of the 400-m track at the fastest speed. ***10 m × 4 round-trip run*:** The test participants ran four times on a 10-m straight track and pushed against wooden blocks placed on both sides of the line. ***Sitting forward bending*:** The test participants sat on the instrument, with their legs straight, and gradually bent their upper body forward and gently pushed the ruler forward with both their hands (no sudden forward movement) until it could not continue to move forward. ***30 s of straight punching sandbags*:** The number of times the participants hit the electronic sandbag with a straight punch over the threshold within 30 s was recorded. ***30 s of kicking sandbags*:** The number of times the participants kicked the electronic sandbag over the threshold within 30 s was recorded. ***30 s of wrestling the dummy*:** The number of falls of the dummy when hit by the participants within 30 s was recorded (caused the dummy to have an apparent head-down fall each time). ***30 s of hitting sandbags using the short baton*:** The number of times the participants hit the electronic sandbags with a short baton over the threshold within 30 s was recorded. ***30 s of poking sandbags using the long baton*:** The number of times the participants poked the electronic sandbags with a long baton over the threshold within 30 s was recorded. ***30 s of pushing sandbags using the shield*:** The number of times the participants pushed the electronic sandbags with their shields over the threshold within 30 s was recorded. ***30 s of trigger pulling according to the gun linkage:*** The number of times the participants pulled the trigger with their forefinger in 30 s was recorded (without bullets).

### Statistical analysis

The test data were statistically analyzed using a Statistical Package for Social Sciences (IBM, SPSS Statistics 25). Factor analysis (principal component analysis) was used for the screening and weighting of indicators; the screening of typical indicators referred to the factor loadings in the principal components; the internal consistency reliability was calculated using Cronbach’s α coefficient; the validity was tested using convergent and differential validity; the evaluation criteria were developed using the percentile rank method; and the validity and accuracy of the standard were calculated using frequency statistics and the chi-squared test.

#### Factor analysis of police physical fitness indicators

To test the suitability of factor analysis for different evaluation items of police physical fitness, KMO (Kaiser–Meyer–Olkin) and Bartlett’s sphericity tests were performed on the test sample data. It is generally considered that the data are suitable for factor analysis when the KMO value is close to 1. The KMO value of our data was 0.825 with a significance of *p* <0.01, which indicated that the data were suitable for factor analysis.

#### Determination of the weight of the police physical quality index

According to the contribution of the factor to the total explained variance of each individual indicator, the weight coefficient of each factor is calculated to obtain the primary indicator weight (Table 2). The weight of each individual indicator in the primary indicator is calculated to combine the absolute value of the eigenvalue of each test indicator in each factor (Table 3), and the weight of each indicator in each factor is calculated according to Equation 3. Finally, multiplying the weight of the indicator in the factor with the weight index of the factor, which is the summation Equation 4, yields the indicator weight model (Kwon, 2011). See Table 4 for details about the weights of the police physical evaluation indicators in each factor.

$$t_{i}=\frac{b_{i}}{\sum_{j=1}^{k}by},$$ (3)

where *t*_*i*_ is the weight of the indicator in the factor, *b*_*i*_ is the absolute value of the eigenvalue of the indicator in the factor, and $\sum_{j=1}^{k}by$ is the sum of the absolute values of the eigenvalues of all indicators.

$$T_{i}=\sum_{j=1}^{k}\left(m_{ij}\times n_{ij}\right),$$ (4)

**TABLE 2:** Weights of physical evaluation indicators for police officers.

Factor name	Indicator	Weight
Upper limb counter quality factor	30 s of trigger pulling according to the gun	0.404
	30 s of straight punching the sandbag	
	30 s of wrestling the dummy	
	Push-ups	
Lower limb counter quality factor	50-m run	0.382
	10 m × 4 round-trip run	
	Standing long jump	
	2,000-m run	
Police confrontation quality factor	30 s of hitting the sandbag using the short baton	0.214
	30 s of poking the sandbag using the long baton	

**TABLE 3:** Score coefficient matrix of physical evaluation components of police officers.

Indicator	Component 1	Component 2	Component 3
30 s of trigger pulling according to the gun	0.377	0.095	−0.066
30 s of straight punching the sandbag	0.362	0.095	−0.011
30 s of wrestling the dummy	0.317	0.032	−0.133
Push-ups	0.240	0.016	0.033
50-m run	0.086	0.387	0.072
10 m × 4 round-trip runs	0.062	0.338	0.035
Standing long jump	−0.127	−0.326	0.114
2,000-m run	−0.039	0.281	0.148
30 s of hitting the sandbag using the short baton	−0.052	0.050	0.566
30 s of poking the sandbag using the long baton	−0.111	0.071	0.661

**TABLE 4:** Weights of police physical evaluation indicators in each factor.

Serial number	Factor name	Indicator	Weight
1	Upper limb counter quality factor	30 s of trigger pulling according to the gun	0.115
		30 s of straight punching the sandbag	0.105
		30 s of wrestling the dummy	0.095
		Push-ups	0.062
2	Lower limb counter quality factor	50-m run	0.115
		10 m × 4 round-trip run	0.095
		Standing long jump	0.116
		2000-m run	0.090
3	Police confrontation quality factor	30 s of hitting the sandbag using the short baton	0.089
		30 s of poking the sandbag using the long baton	0.118

where *T*_*i*_ is the weight of the indicator, *m*_*ij*_ is the weight index of the factor, and *n*_*ij*_ is the weight of the indicator in the factor.

#### Construction of a reliability test of police physical fitness

One-factor correlation analysis was used to test the reliability of the basic structural factors of police physical quality based on the real-world orientation.

#### Construction of a validity test of police physical fitness

Multi-factor correlation analysis was used to test the validity of the factor constructs of the basic physical quality of police fitness based on real-world combat.

#### Standard values for police physical fitness scores

Using the scoring criteria developed by Wenhua (2008), the data of each test index were transformed into a corresponding score according to the percentile position (P90, P80, and P70–P10). The transformed scores were multiplied by the weight of the index and then summed to obtain the comprehensive evaluation score of police physical quality. The scores of each individual index are shown in Table 5.

**TABLE 5:** Contrast table of the individual and comprehensive scores of police officers.

Grade	Percentile (%)	Score
Excellent	≥90	100
Good	70–90	80
Medium	30–70	60
Qualified	10–30	40
Poor	<10	20

#### Standard values for the comprehensive evaluation of police physical fitness

To objectively reflect the variability of different levels of police physical quality in China, comprehensive evaluation standard values were established based on the individual evaluation standard values. The method for developing the comprehensive evaluation standard values was a three-step process: 1) using the single-scoring table of each typical index of the whole sample, the measured data of the tested police officers were converted into scores according to the evaluation standard values (Table 6). 2) The scores of the tested police officers were calculated separately, and the weighted score of each index was obtained by multiplying the score of each index by its weight in the index of that level. The weighted scores were then summed to generate the comprehensive evaluation score of the physical fitness of the tested police officers. 3) Finally, the P90, P70, P30, and P10 percentiles of the total sample scores were calculated to obtain the comprehensive evaluation standard value (Xing, 1985). Table 7 shows the details of the comprehensive evaluation standard values.

**TABLE 6:** Standard values for the individual evaluation of police physical fitness based on law enforcement ability.

Individual evaluation indicator	Excellent	Good	Medium	Qualified	Poor
30 s of trigger pulling according to the gun (*n*)	≥56	47–55	33–46	24–32	≤23
30 s of straight punching the sandbag (*n*)	≥54	43–53	28–42	18–27	≤17
30 s of wrestling the dummy (*n*)	≥10	8–9	6–7	4–5	≤3
Push-ups (*n*)	≥46	40–45	31–39	25–30	≤24
50-m run (time, s)	≤7.3	7.4–7.7	7.8–8.3	8.4–8.9	≥9.0
10 m × 4 round-trip run (time, s)	≤10.2	10.3–10.8	10.9–11.8	11.9–12.8	≥12.9
Standing long jump (m)	≥2.39	2.25–2.38	2.07–2.24	1.95–2.06	≤1.94
2000-m run (time, min + s)	≤8.39	8.40–9.33	9.34–10.50	10.51–11.46	≥11.47
30 s of hitting the sandbag using the short baton (*n*)	≥34	26–33	14–25	10–13	≤9
30 s of poking the sandbag using the long baton (*n*)	≥35	27–34	18–26	11–17	≤10

*n*, number of times; s, seconds; min, minute; m, meter.

**TABLE 7:** Comprehensive standard values of police physical fitness based on law enforcement ability.

Classification standard values (percentile, %)	Overall rating (score)	Grade
≥90	>62.27	Excellent
70–90	53.56–62.26	Good
30–70	42.40–53.55	Medium
10–30	34.72–42.39	Qualified
<10	<34.71	Poor

#### Back test of comprehensive evaluation standard values

Next, the accuracy and validity of the standard values developed for the evaluation of police physical quality were verified.

## Results

### Construction of a police physical quality evaluation model based on law enforcement

The evaluation indexes were analyzed by factor loadings, and the results showed that the factor loadings of sitting forward bending (0.301), 30 s of hitting the sandbag with a stomp (0.337), and 30 s of pushing the sandbag with a shield (0.419) were significantly lower than the high-load-factor criteria (loadings >0.5); thus, these three indicators were eliminated in agreement with the expert opinions. The factor loading of 30 s of hitting the sandbag using a short baton (0.452) was close to the expert opinion, and this indicator was retained owing to its police occupational specificity. Therefore, push-ups (0.536), standing long jump (0.642), 50-m run (0.775), 10 m × 4 round-trip run (0.666), 2,000-m run (0.535), 30 s of straight punching the sandbag (0.825), 30 s of wrestling the dummy (0.543), 30 s of hitting the sandbag using the short baton (0.452), 30 s of poking the sandbag using the long baton (0.624), 30 s of hitting the sandbag using the short baton (0.624), and 30 s of trigger pulling according to the gun linkage (0.782) were established as 10 indicators of police physical fitness.

The results of the total explained variance of the police physical quality evaluation indexes revealed that the initial eigenvalue was 1.109 and the cumulative contribution of the three main factors was 67.739%. Therefore, the first three factors could be accepted as the main constituents of police physical quality under real-world conditions (Table 8). To make the study of these three factors more practical, factor orthogonal rotation was performed on the three factors, and the factor loadings of each indicator with loadings >0.5 on each factor after rotation were obtained (Table 9).

**TABLE 8:** Total explanatory variance of the police physical evaluation index based on law enforcement ability.

Serial number	Initial eigenvalue			Extraction of the sum of the square of loads			Sum of the squared rotating loads		
	Total	Variance %/%	Cumulative %	Total	Variance %/%	Cumulative %	Total	Variance %/%	Cumulative %
1	4.069	40.689	40.689	4.069	40.689	40.689	2.735	27.351	27.351
2	1.596	15.959	56.648	1.596	15.959	56.648	2.589	25.887	53.238
3	1.109	11.091	67.739	1.109	11.091	67.739	1.450	14.500	67.739
4	.654	6.542	74.280						
5	.639	6.393	80.674						
6	.564	5.636	86.310						
7	.461	4.612	90.921						
8	.419	4.190	95.111						
9	.312	3.115	98.226						
10	.177	1.774	100.000						

**TABLE 9:** Factor load of the police physical evaluation index after rotation.

Indicator	Factor 1	Factor 2	Factor 3
30 s of trigger pulling according to the gun	0.881	—	—
30 s of straight punching the sandbag	0.881	—	—
30 s of wrestling the dummy	0.737	—	—
Push-ups	0.664	—	—
50-m run	—	0.868	—
10 m × 4 round-trip run	—	0.789	—
Standing long jump	—	−0.774	—
2000-m run	—	0.683	—
30 s of poking the sandbag using the long baton	—	—	0.837
30 s of hitting the sandbag using the short baton	—	—	0.754

The absolute value of each indicator in the factor analysis and expert opinions was used to determine the attribution of indicators to each factor, and the factors were subsequently named. Four indicators—30 s of trigger pulling according to the gun, 30 s of straight punching the sandbag, 30 s of wrestling the dummy, and push-ups—constituted the first factor, which was named the upper limb confrontation quality factor. Another four indicators—50-m running, 10 m × 4 round-trip running, standing long jump, and 2,000-m running—constituted the second factor, which was named the lower limb confrontation quality factor. In addition, 30 s of poking sandbags using the long baton and 30 s of hitting sandbags using the short baton were the two indicators that constituted the third factor, which was named the police equipment confrontation quality factor.

#### Completing the construction of the police physical fitness model

As a result, a combat-oriented police physical fitness evaluation model is derived: Y = 0.115 × 30 s of trigger pulling according to the gun + 0.105 × 30 s of straight punching the sandbag + 0.095 × 30 s of wrestling the dummy + 0.062 × push-ups + 0.115 × 50-m run + 0.095 × (10 m × 4 round-trip run) + 0.116 × standing long jump + 0.090 × 2,000-m run + 0.089 × 30 s of hitting the sandbag using the short baton + 0.118 × 30 s of poking the sandbag using the long baton. These results are presented in Table 4.

The results showed that the correlations of all indicators were greater than 0.3; the internal consistency Cronbach’s coefficients were >0.5, including 0.784 for upper limb confrontation quality, 0.568 for lower limb confrontation quality, and 0.539 for police weapon confrontation quality.

The results showed that the research factor constructs were divided into three parts: upper limb confrontation quality, lower limb confrontation quality, and police weapon confrontation quality. The convergent validity of each factor construct ranged from 0.610 to 0.634, which was an acceptable range, and the correlation coefficients ranged from 0.2 to 0.7, which was not too high or too low.

### Establishment of standards for police physical fitness scores

The raw data of the tested police officers at different levels were converted into comprehensive physical quality scores according to the evaluation standard values and weights of each index, and frequency statistics and chi-squared tests were performed according to the comprehensive scores at different levels (Tables 10, 11). There was a highly significant difference (*p* <0.01) between the ordinary police officers (“general” group) and police officers participating in the competition (“excellent” group).

**TABLE 10:** Frequency statistics of the comprehensive evaluation for police physical ability at different levels.

Evaluation level	Tournament police (“excellent” group)	General police (“general” group)	Total
Excellent	25	5	30
Good	3	57	60
Medium	0	121	121
Down	0	60	60
Difference	0	30	30
Total	28	273	301

**TABLE 11:** Chi-squared test statistics of the comprehensive standard value of the physical ability of police officers of different ranks.

	Value	Degree of freedom	Progressive significance (bilateral)
Pearson’s cardinal	226.295a	4	0.000
Likelihood ratio (L)	137.140	4	0.000
Linear correlation	91.681	1	0.000

## Discussion

Police law enforcement is unpredictable and physically challenging. In this study, we showed that upper limb confrontation, lower limb confrontation, and confrontation with the use of police equipment are the possible forms of confrontation faced by police officers and that these three factors constitute a combat-oriented police physical fitness model. The indicators covered by the factors are mutually dependent on each other and reflect the significant characteristics in winning law enforcement battles. On the basis of their large factor loadings, close connection with police combat, and facileness, the indicators from each principal component were selected as representatives. Some of the basic physical evaluation indicators in this study have been mentioned in other studies, such as push-ups (Marins et al., 2019), standing long jump (Tian Wen, 2008), and 50-m run (Dong Rujun, 1999; Araújo et al., 2017). Some indicators such as 10 m × four round-trip run and 2,000-m run are the physical fitness test indexes of the Chinese police; other specific fitness indicators are derived from innovations in this study. Table 9 shows that the established factor analysis model is appropriate, and the selected indicators fully represent the characteristics of police physical quality based on the law enforcement ability orientation (Guo Zhong and Yingqiu, 2004). The reliability of the basic structural factors of police physical quality met the statistical requirements (Wu et al., 2019). In addition, the open-root values of the diagonal AVEs were all greater than the correlations between the other factor constructs and the factor constructs, representing there is a differential validity between factor constructs (Wu et al., 2019) (Table 12). Therefore, the constructed factor validity of police physical fitness-based constructs meets the statistical requirements.

**TABLE 12:** Validity test analysis of different factors.

—	Convergent validity	Differential validity			Descriptive statistics		
	AVE	Upper limb	Lower limb	Police arms	Average value	Standard deviation	Case (*n*)
Upper limb	0.633	0.796	—	—	118.226	31.047	301
Lower limb	0.610	−0.406	0.781	—	31.591	2.325	301
Police arms	0.634	0.376	−0.232	0.796	43.973	15.795	301

Upper body confrontation quality indicators include push-ups, straight punches, wrestling dummies, and trigger pulling based on gun linkage. Push-ups are a common means of testing the upper body and waist and abdominal strength quality of police officers and are widely used in various countries because this test method is simple, not restricted by the venue, and can cover police officers of all ages (Marins et al., 2019). Police law enforcement tasks such as pushing/pulling suspects and shoving vehicles are common actions that require a strong upper body and waist and abdominal strength as support. Straight punch is an effective unarmed countermeasure, and its technical method can be easily mastered by frontline police officers and can be used to evaluate the special quality of unarmed combat skills (Nieuwenhuys et al., 2009). In accordance with the law enforcement requirements of force-level equivalence, in the face of unarmed attacks by suspects, police officers need to fight back in an unarmed manner. The 30 s of straight punching sandbags, in the premise of setting the threshold for striking, requires a certain striking force, but also emphasizes the striking speed, so as to ensure that police officers have a self-defense counterattack ability in the event of an attack. Wrestling dummies is an important part of police unarmed defense and control or grappling training. It can be used to train police officers in the physical quality of wrestling control ability because many of the professional requirements of police work involve the body control of suspects (Anderson et al., 2001). Police arrests in law enforcement combat are more commonly used to control the fall, such as double full-confrontation wrestling, full control, and counter-control after the fall (Fu and Jia, 2021), which requires police officers to have a certain ability to control the fall, in order to achieve the training purpose of catching and firm control. Gun linkage trigger pulling can be used for pistol aiming and firing simulation training. The nine-two pistol, which the police in China are equipped with, is a general-purpose pistol that has a linkage firing trigger buckle pressure of 30 s. The gun linkage trigger buckle reflects the stability of the arm according to the aim of the gun and fast buckling of the finger when shooting at the target to ensure the accuracy of the final hit.

Lower limb confrontation quality indicators include standing long jump, 50-m run, 10 m × 4 round-trip run, and 2,000-m run. Standing long jump is mainly an indicator to measure the explosive power of lower limb muscles, bouncing power, and body coordination ability when the human body jumps forward horizontally and is commonly used in fitness tests for the general population and police professionals (Tian Wen, 2008). Police officers often have to jump obstacles in the process of chasing suspects, which puts certain requirements on the explosive power of the lower limbs (Lonsway, 2003). The 50-m run can effectively measure the ability of the human body to move quickly and is often used as a police speed test item (Dong Rujun, 1999; Araújo et al., 2017). The rescue of members of the public and the pursuit and arrest of suspects are part of the routine work of the police (Lockie et al., 2018), and in such situations, the police on duty are required to have an ability to sprint short distances. The 10 m × 4 round-trip run can reflect the test subject’s speed of action, agility, and coordination ability. By incorporating a start, acceleration, emergency stop (touch markers), sharp turn, and other links, the 10 m × 4 round-trip run can effectively measure the ability of the police to sprint, emergency stop, turn sharply, bend, squat, and fold (Anderson et al., 2001). The 2,000-m run was proposed as the endurance part of China’s all-police combat training, following an all-police, all-age mandatory test project that drew on the distance covered in 12 min of running at home and abroad. Owing to the high intensity and duration of police law enforcement work, endurance training has become a vital element of the training content to support the police combat work.

Police confrontation quality indicators include strikes using a short baton and pokes using a long baton. Short batons (i.e., telescopic batons) are an essential police weapon issued to police officers domestically and in other countries and can be used as an offensive and defensive means for police officers to protect themselves and fight criminals. Strikes using a short baton can be used as a police weapon for arrests and self-defense skills of special quality (Nieuwenhuys et al., 2009). In terms of the type of strikes, the hitting technique is easily mastered by frontline police officers. The test item of 30 s of hitting the sandbag using the short baton requires the tested police officers to have a certain hitting power and a certain hitting speed under the premise of mastering technical movements, thereby training their ability to use short-baton striking. A long baton (i.e., riot baton) can be used to pull away from the suspect in case of extreme crimes of violent attacks with knives. The distance from the suspect ensures the police officer’s own safety in such situations. Long-baton poking requires a strong and small action range, a strong hand in the back, rapid poking and stabbing after retrieval, and consistent striking and sprinting to achieve fast, accurate, and hard strikes (Huang Zijian, 2018). In addition, long-baton poking can be used as a unique quality indicator of police equipment use.

In previous studies, police physical fitness evaluations were mostly focused on basic physical fitness indicators, such as general strength, endurance, and speed (Dawes et al., 2021), or simulated professional task combination-based physical fitness evaluations (Orr et al., 2022); however, there was a lack of specialized physical fitness indicators based on law enforcement ability characteristics. Furthermore, there is no established evaluation model for police physical fitness. This study conducts an integrated assessment of physical skills based on the characteristics of police law enforcement and equipment carried or used by the police. It constructs a physical fitness assessment model based on law enforcement capabilities and the corresponding evaluation standards, providing new ideas for police physical fitness training.

## Limitation

There are certain study limitations that should be acknowledged. The authors have not included the statistics on the physical fitness levels of the participants, which may have influenced the results of the study. In addition, all police subjects were male, and further exploration may be needed to construct a physical fitness model for female officers in the future.

## Conclusion

In conclusion, this study analyzes the evaluation indexes of police physical quality based on law enforcement: push-ups, straight punches, wrestling dummies, trigger pulling according to the gun linkage, standing long jump, 50-m run, 2,000-m run, 10 m × 4 round-trip run, short-baton hitting, and long-baton poking. An evaluation model of police physical quality based on law enforcement can be constructed as follows: Y = 0.115 × 30 s of trigger pulling according to the gun + 0.105 × 30 s of straight punching the sandbag + 0.095 × 30 s of wrestling a dummy + 0.062 × push-ups + 0.115 × 50-m run + 0.095 × (10 m × 4 round-trip run) + 0.116 × standing long jump + 0.090 × 2,000-m run + 0.089 × 30 s of hitting the sandbag using the short baton + 0.118 × 30 s of poking the sandbag using the long baton. The standard values of the police physical fitness evaluation criteria established by the back generation test evaluation standard values are objective and valid.

## Practical application

There are several practical applications that can be drawn from this research. Police physical training should consider the equipment carried by the police, and the skills of using the equipment should be taken into account when providing physical fitness training. This evaluation model and standards are applicable to the training, assessment, and competition of frontline law enforcement officers on duty. Further research may be needed for SWAT and female police officers, who are currently not suitable for this standard.

## Data Availability

The raw data supporting the conclusion of this article will be made available by the authors without undue reservation.
